# Clearing the slate: RNA turnover to enable cell state switching?

**DOI:** 10.1242/dev.202084

**Published:** 2023-10-12

**Authors:** Elizabeth R. Westbrook, Hugh Z. Ford, Vlatka Antolović, Jonathan R. Chubb

**Affiliations:** UCL Laboratory for Molecular Cell Biology, University College London, Gower Street, London WC1E 6BT, UK

**Keywords:** RNA decay, Cell state transition, Ribosome competition

## Abstract

The distribution of mRNA in tissue is determined by the balance between transcription and decay. Understanding the control of RNA decay during development has been somewhat neglected compared with transcriptional control. Here, we explore the potential for mRNA decay to trigger rapid cell state transitions during development, comparing a bistable switch model of cell state conversion with experimental evidence from different developmental systems. We also consider another potential role for large-scale RNA decay that has emerged from studies of stress-induced cell state transitions, in which removal of mRNA unblocks the translation machinery to prioritise the synthesis of proteins that establish the new cell state.

## Teaser

**Summary:** Large-scale RNA turnover is a recurring feature of cell state transitions in development. In this Spotlight, we consider the roles of turnover in cell state destabilisation and in establishing the subsequent developmental stage.

## Introduction

The snapshots of RNA expression that still dominate how we characterise gene regulation in embryos have somehow become a proxy for transcription. This is not completely unjustified, since the embryo needs the RNA to be made, but it overlooks the fact that the amount of RNA present in a cell is a balance between its synthesis and its removal. Indeed, the importance of RNA turnover, together with transcription, for shaping transcript levels during development has long been established (Alonso, 2012; Bashirullah et al., 2001). It is also important to consider RNA turnover as a control strategy in its own right, since this is a potentially powerful mechanism for enabling rapid switching between gene expression states. Changing cell state solely using transcriptional control is time consuming; the cell must signal to the gene in question, assemble a variety of complexes around the gene, engage a polymerase, initiate the polymerase, then wait for clearance to bypass any pauses before elongation can begin. The nascent mRNA must then undergo termination, polyadenylation, capping, splicing, export and localisation, before translation takes place. This, in turn, is followed by protein folding, localisation, modification and assembly. In contrast, although mRNAs can persist for timescales of up to days, RNA turnover, once initiated, is so efficient that decay intermediates for even highly abundant transcripts can be difficult to detect (Houseley and Tollervey, 2009). This efficiency is perhaps, in part, due to the redundancy between different turnover pathways and the high processivity of some of the enzymatic components (Horvathova et al., 2017). Using RNA decay, a change in the transcriptome can be effected in seconds to minutes, rather than tens of minutes to hours (Van Haastert et al., 1992).

In this article, with the aid of a standard mathematical framework for cell state conversions, we explore how abrupt changes in transcript content by mRNA decay might enable rapid and coherent changes of cell state during developmental progression, and, conversely, how increasing transcript stability may stabilise existing cell states. We also explore an alternative potential benefit of extensive transcript clearance that has emerged from studies of stress-induced cell state transitions (Bercovich-Kinori et al., 2016; Lackner et al., 2012; Lee et al., 2011) in which turnover frees the translation machinery to prioritise new transcripts. We do not discuss RNA turnover mechanisms in detail here, since these have already been covered extensively (Houseley and Tollervey, 2009). Overall, we hope to reignite discussion around the importance and functions of RNA turnover in developmental transitions.

## RNA turnover to destabilise cell states

In rapidly changing embryonic contexts, the potential to quickly change the transcriptome by RNA destruction may be essential. If transcripts from an early state are retained during developmental progression, the cell would potentially retain responsiveness to signals promoting the early state. This would be challenging for cells in the complex signalling environments of tissues, particularly for migrating cells, which change their signalling environments over time. An incoherent or dissonant cell state might be useful during developmental decision making in which cells continually seek information from their surroundings to evaluate their decision process. It would not be so advantageous for a quick or irreversible step. Conversely, even if a cell is exposed to strong signalling promoting a new state, the cellular phenotype may remain locked in the initial state if transcript (and protein) turnover is not part of the cell response. Testing these potential regulatory scenarios for turnover is limited by the experimental resolution possible for investigating such highly redundant and complex control systems, although it is abundantly clear from perturbation studies in many systems that RNA turnover is a crucial feature of developmental progression (Alonso, 2012). Faced with this complexity, mathematical models can be useful to orient our understanding.

**Fig. 1.**
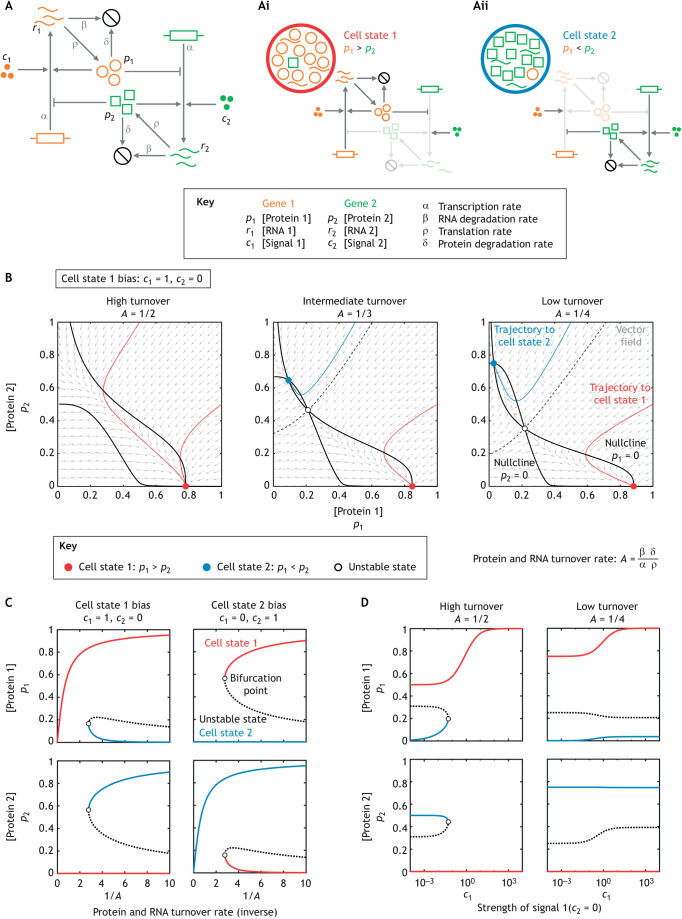
**Outline and analysis of a simple mathematical model of an RNA turnover-induced bistable switch in cell states.** (A) Model schematic. Two genes encode proteins that promote the transcription of their own RNA species while inhibiting the other. Orange and green elements represent the components for gene 1 and 2, respectively. Grey symbols represent the rates governing the reactions. For example, the quantity of each RNA species (*r*_1_ and *r*_2_) decreases via degradation at rate *β* (proportional to RNA quantity). The quantity of each protein species (*p*_1_ and *p*_2_) increases via translation at rate *ρ* (proportional to the quantity of RNA) and decreases via degradation at rate *δ* (proportional to protein quantity). Protein levels promote their own expression and repress expression of the other gene. This activation and repression is modelled using Hill functions. Model details can be accessed from Chubb and Ford (2023). (Ai,Aii) This system can exist in two stable states defined by different values of *p*_1_ (Ai) or *p*_2_ (Aii), with components at high levels (‘On’) in dark grey and those at low levels (‘Off’) in light grey. (B) Perturbing RNA turnover in the model uses the parameter *A*, the turnover rate, which aggregates the synthesis and degradation rates of transcript and protein. Shown are phase plots with a signalling bias towards cell state 1 (*c*_1_=1, *c*_2_=0) at different turnover rates. Red and blue circles represent stable states associated with cell state 1 and 2, respectively; the white circle represents the unstable state. The vector field summarises how *p*_1_ and *p*_2_ change given their current values. Nullclines are shown as solid black lines that represent where either *p*_1_ or *p*_2_ do not change. Trajectories, which reflect how the cell states change over time, are shown as solid lines coloured by the stable state to which they converge. The dashed line is the boundary between the basins of attraction of the stable states. At low turnover, both stable states exist. At high turnover, there is one stable state (cell state 1) favoured by the signal. (C,D) Bifurcation plots of the steady state values of *p*_1_ (top row) and *p*_2_ (bottom row) with respect to the inverse turnover rate (C) and strength of signal 1, *c*_1_ (when signal 2, *c*_2_=0) (D). Cell state 1 and 2 are coloured red and blue, respectively. The lines show the quantity of protein associated with each state. The unstable state is shown as a dotted black line. White circles represent bifurcation points, which is where the turnover rate (C) or signal strength (D) separate regimes in which there exist either one or two potential cell states.

Simple models of minimal gene regulatory networks have been used to simulate a variety of cell state transitions (Alon, 2007), usually addressing the effects of transcription factor concentration or activity rather than degradation processes. Here, we leverage a standard two-gene modelling framework to illustrate how RNA degradation can affect cell state switching (Chubb and Ford, 2023). The model shows that low mRNA turnover rates allow a cell state to remain stable even in the presence of signals promoting another state, with increasing RNA turnover a potent mechanism for initiating cell state switching. In the model, proteins (quantities denoted by *p*_1_ and *p*_2_) self-activate their own transcription while mutually repressing each other (Fig. 1A). This generates two stable cell states: one in which *p*_1_ is greater than *p*_2_ (cell state 1; Fig. 1Ai) and one in which *p*_2_ is greater than *p*_1_ (cell state 2; Fig. 1Aii). The external signals promoting each state (quantities denoted by *c*_1_ and *c*_2_) are independent variables that induce the transcription of genes 1 and 2 with the same potency as protein 1 and 2, respectively. The dynamics of the overall system are governed by one parameter, *A*, which aggregates the synthesis and degradation rates of both transcripts and proteins (Fig. 1B). We can explore the system in different signalling regimes (Fig. 1B). For example, if *c*_1_ is greater than *c*_2_, then at low turnover rates (*A*=1/4, right), the system can exist in either cell state 1 (red circle) or 2 (blue circle), despite the biased signalling, because high levels of protein 2 repress expression of gene 1. This situation changes for intermediate and higher mRNA turnover rates: cell state 2 destabilises, converging towards an unstable cell state (white circle; Fig. 1B, middle) and completely disappears such that only cell state 1 is stable (Fig. 1B, left). In other words, stable mRNA allows the persistence of a state not favoured by the signalling environment. A burst of non-specific mRNA (or protein) decay is sufficient for the system to then switch to the state favoured by the signalling. Comprehensive exploration of different signalling weights (Fig. 1C) and RNA turnover weights (Fig. 1D) supports this conclusion; this is exemplified by the bottom right panel in Fig. 1D, in which cell state 2 (blue line) is completely insensitive to signal *c*_1_ at any strength, as long as RNA turnover is low.

Although this is only a model, several lines of evidence suggest that the model points us in a reasonable direction. The early embryo of *Drosophila* gives us some clear examples. Early zygotic transcripts can have very short half-lives (a few minutes only) (Edgar et al., 1986). This may be essential for the remodelling of their expression domains. This perhaps matters most for transcripts, such as *ftz*, encoding proteins that provide positive feedback to their own transcription and negative feedback to other genes. *ftz* mRNA is initially expressed as a continuous band before transitioning to a regular striped pattern (Edgar et al., 1986). Without rapid turnover, the ability of Ftz protein to stimulate *ftz* transcription (Birnie et al., 2023; Zhao et al., 2023) would prevent the restriction of the pattern to stripes. More generally, extensive degradation of maternal mRNA in animal embryos occurs to clear the slate for zygotic gene expression (Schier, 2007). Mutants with strong defects in maternal RNA clearance arrest developmental progression before large-scale zygotic expression begins (Tadros et al., 2007). RNA turnover may be a key element of a broader range of sharp cell state transitions than maternal-to-zygotic transitions (MZTs), with single-cell studies in non-animal differentiation systems showing rapid transitions between discrete gene expression states that are characterised by substantial transcript loss events (Antolovic et al., 2019; Nelms and Walbot, 2019).

One feature of these examples not considered by the model is transcript specificity – not all transcripts are subject to the same global regulatory rules (Rabani et al., 2017) and the removal of specific transcripts may drive developmental transitions. An additional consideration is how the decay response is set or induced by developmental signalling. Our understanding of links between signalling and RNA decay is sparse in most developmental contexts. This is perhaps due to the complexity of both the signalling and decay machineries and their requirement for housekeeping duties, together with difficulties in cleanly measuring and manipulating both signalling and decay in heterogeneous cell populations. *In vitro* differentiation models have been used as an entry point into this challenge, by facilitating the analysis of RNA-binding protein and RNA modification dynamics, and measurement of RNA turnover kinetics (through blocking transcription or metabolic labelling) in accessible and relatively homogenous cell populations (Batista et al., 2014; Geula et al., 2015). Indeed, we can find support for the ‘clearing the slate’ model in studies of the abundant *N*^6^-methyladenosine (m6A) RNA modification in embryonic stem cells (ESCs). Inhibiting the enzymes that catalyse the addition of this RNA modification increases the stability of many RNAs, including pluripotency regulators, and impairs differentiation (Batista et al., 2014; Geula et al., 2015). Impaired differentiation of stem cells in mutants that exhibit stabilised RNAs is also observed in Planaria (Solana et al., 2013). However, we can also find evidence from ESCs that is not immediately consistent with the slate-clearing model. For example, m6A is also implicated in stabilising pluripotency (Wang et al., 2014), and other perturbations of RNA turnover, such as inhibition of the deadenylase component CNOT3 (Zheng et al., 2016), suggest that RNA decay prevents the emergence of the differentiating state. The idealised narrative here, of a burst of RNA turnover removing pluripotency regulators to destabilise the cell state, does not emerge clearly from these initial studies, although the overall interpretation of effects may be clouded by the low resolution of genetic approaches to study global regulators. Some clarity perhaps emerges from long-term single-cell imaging of fluorescent pluripotency reporters. Monitoring the dynamics of these protein reporters over multiple cell generations reveals that m6A depletion reduces the reversion of cells primed for differentiation back to pluripotency (Jin et al., 2021). This implies stabilisation of the emerging differentiating state by depletion of m6A, in addition to any effects on pluripotency. These data remind us of observations early in the molecular biology age in *Dictyostelium*, in which cells specifically reduce the half-life of developmental transcripts upon the induction of de-differentiation (Chung et al., 1981). This scenario, of stable RNA stabilising cell states and unstable RNA destabilising them, is very much in line with the model in Fig. 1. Indeed, selective stabilisation of maternally encoded transcripts, in addition to any transcriptional control, may contribute to the stabilisation of specific cell states (germ cells, for example) through the otherwise extensive RNA decay occurring during the MZT (Fishman et al., 2023 preprint).

## RNA turnover for translational resource reallocation

The model may appeal, but considering RNA in isolation misses an important point. Clearing the mRNA slate might be required, but the idea that it is necessary for the removal of an old cell state surely also depends on what happens to the proteins that the degraded RNAs would have encoded. Combining dynamic measurements of RNA and protein turnover in complex animal tissues is not a trivial task. This approach has, however, been carried out for a variety of cell state transitions in response to environmental perturbations in single-cell organisms (Lackner et al., 2012; Lee et al., 2011). Although conflating cell state transitions in these stress responses with cell state transitions in development is something not all developmental biologists are comfortable with, it is worth considering that many (if not most) non-animal differentiation events are reactions to external stimuli, and these systems make use of much of the same signalling and core RNA degradation machinery as animal developmental systems (Houseley and Tollervey, 2009).

These studies suggest that clearing the slate may have other benefits. Monitoring both transcriptome and proteome changes in yeast after different types of stress (such as oxidative stress and osmotic stress) suggests that although loss of transcripts is a significant part of stress-associated transcriptome remodelling, transcript changes are not necessarily reflected in a decline in abundance of the respective proteins (Lackner et al., 2012; Lee et al., 2011). Upregulated transcripts are correlated with an increase in the abundance of their associated proteins, consistent with the need to adapt to a new state, yet downregulated transcripts show no substantial decrease in the expression of the proteins they encode. The latter finding indicates that in some cell state transitions with substantial transcript loss, it is not the loss of the proteins these transcripts encode that triggers the change in cell behaviour. In a developmental context, protein retention despite transcript loss might allow the cell to ‘hedge its bets’ – exploring a particular fate programme while allowing the possibility to revert to an earlier state if reallocation of fates is required, perhaps in a rapidly changing signalling environment. The absence of large-scale protein loss in these contexts may also be explained by models of translation dynamics suggesting that ribosome availability is a rate-limiting step in the manufacture of proteins (Lee et al., 2011; Shah et al., 2013), meaning the loss of transcripts would free translational resources (ribosomes, tRNA, amino acids) for the new proteins required. Translational resource reallocation is also present during cell state transitions that accompany viral infections. Viruses can hijack the host RNA-degradation machinery to target host transcripts for degradation (Gaglia et al., 2012). This is specific to RNA Polymerase II (RNA Pol II) transcripts, as RNAs produced by Pol I or Pol III are not degraded upon exposure to viral components (Gaglia et al., 2012). Knockouts of components of the host degradation machinery reduce or completely stop infection (Garcia-Moreno et al., 2019). This selective degradation would free the translational machinery to favour synthesis of viral proteins over proteins from the host cell, in addition to restricting the production of antiviral components (Bercovich-Kinori et al., 2016).

To what extent can these insights into cell state transitions from stressed cells be extrapolated to developmental contexts? The data are sparse, perhaps due to the standard developmental biology narratives based on explaining the effects (developmental arrest) of a perturbation (blocking RNA turnover) on known components (such as pluripotency factors). However, the conclusions from recent quantitative proteomics data spanning the *Drosophila* MZT may not be so dissimilar to those from the stress response data: although around 60% of maternally encoded transcripts are degraded in the early embryo, only 2% of maternal proteins show a significant decrease (Cao et al., 2020). As far as the cell state is concerned, this is hardly ‘clearing the slate’. In mouse ESCs, data on the RNA-binding protein CAPRIN1 are also consistent with the data from cell-stress responses. *Caprin1* knockout ESCs show defects during early differentiation, an effect associated with an increase in the half-lives of thousands of transcripts in both undifferentiated and differentiating cells (Viegas et al., 2022). CAPRIN1 protein interacts extensively with the m6A machinery, although its RNA turnover role is mediated, at least in part, by the 5′-3′ exoribonuclease XRN2 (Viegas et al., 2022). The many transcripts that change stability in the *Caprin1* knockouts (∼2500 in both undifferentiated and differentiation conditions) showed no clear enrichments for specific functions, again implying no coherent clearing of the slate (Viegas et al., 2022). These examples suggest that the degraded transcripts may be conveying no specific information themselves; they are just unwanted transcripts that block the cell state transition when retained, which may relate to a failure to streamline the translation machinery, as in the stress- or virus-induced responses. Along these lines, across a broad range of different stem cell contexts, undifferentiated cells show reduced global translation rates compared with their differentiating progeny (Teixeira and Lehmann, 2019). With translation capacity low, an undifferentiated cell attempting to progress in development would presumably benefit from a burst of RNA degradation to prioritise translation of mRNAs required for the next stage.

RNA clearance may also be employed more passively to enable cell state transitions during development, for example during head-to-tail development in vertebrates. Here, a pulse of transcription of *FGF8* at the tail is followed by cells moving away from the tail zone (Dubrulle and Pourquié, 2004). With no new transcription, degradation of existing *FGF8* mRNA forms a gradient as the cells gradually spread out along the head-to-tail axis, with low FGF8 protein levels further from the tail conducive to somite formation (Dubrulle and Pourquié, 2004). One limitation of this strategy is that low signal levels are likely to be susceptible to noise and so may not be especially reliable at triggering a cell state switch. This consideration suggests that a decay-driven, gradient-based mechanism such as this is likely to be, at most, permissive. Perhaps unsurprisingly, precise somite formation requires the superimposition of multiple additional layers of control (Simsek et al., 2023).

## Perspectives

We have discussed two potentially beneficial roles for ‘clearing the slate’ to facilitate developmental progression. These roles are not mutually exclusive, and it is not trivial to distinguish between these and other potential benefits of transcript clearance, such as resource recycling (Vastenhouw et al., 2019). We have also only considered mRNA. It is striking that many of the embryonic examples discussed here (such as the MZT in flies and some vertebrate models) are in contexts with cell division but minimal cell growth. This minimises the potential for dilution of RNA to contribute to cell state changes, potentially increasing the reliance on more aggressive degradation events.

A functional test of the clearing the slate view would be to take a sharp state transition with a strong RNA turnover component, then precisely stage the turnover event with respect to a change in the functional state of the cells, such as a loss of potential for cells to return to the initial state (‘commitment’). This might involve, for example, imaging the turnover event using a fluorescent RNA-tagging approach (Hu et al., 2023) *in vivo* and administering a treatment promoting the original cell state at varying times in the transition. At which point relative to RNA turnover does the cell state functionally change? Coupling imaging of RNA dynamics with imaging the dynamics of the encoded protein will also be an important part of future explorations of the roles of RNA turnover in cell state transitions.
